# Supplementary material for: Plasmodium falciparum infection in humans and mosquitoes influence natural Anopheline biting behavior and transmission
Source: Nat Commun. 2024 May 30;15:4626. doi: 10.1038/s41467-024-49080-9 (PMC11139876; doi:10.1038/s41467-024-49080-9)
Supplement: Supplementary file 3 — Description of Additional Supplementary Files [file 41467_2024_49080_MOESM3_ESM.pdf]

Description of Additional Supplementary Files

File Name: Supplementary Data 1
Description: Primer sequences for all molecular assays
