# Supplementary material for: Plasmodium falciparum infection in humans and mosquitoes influence natural Anopheline biting behavior and transmission
Source: Nat Commun. 2024 May 30;15:4626. doi: 10.1038/s41467-024-49080-9 (PMC11139876; doi:10.1038/s41467-024-49080-9)
Supplement: Supplementary file 5 — Reporting Summary [file 41467_2024_49080_MOESM5_ESM.pdf]

nature portfolio

Corresponding author(s): Markwalter

Last updated by author(s): 2024 04 11

Reporting Summary

Nature Portfolio wishes to improve the reproducibility of the work that we publish. This form provides structure for consistency and transparency in reporting. For further information on Nature Portfolio policies, see our Editorial Policies and the Editorial Policy Checklist.

Statistics

For all statistical analyses, confirm that the following items are present in the figure legend, table legend, main text, or Methods section.

n/a	Confirmed	
☐	☒	The exact sample size (*n*) for each experimental group/condition, given as a discrete number and unit of measurement
☐	☒	A statement on whether measurements were taken from distinct samples or whether the same sample was measured repeatedly
☐	☒	The statistical test(s) used AND whether they are one- or two-sided<br>*Only common tests should be described solely by name; describe more complex techniques in the Methods section.*
☐	☒	A description of all covariates tested
☐	☒	A description of any assumptions or corrections, such as tests of normality and adjustment for multiple comparisons
☐	☒	A full description of the statistical parameters including central tendency (e.g. means) or other basic estimates (e.g. regression coefficient) AND variation (e.g. standard deviation) or associated estimates of uncertainty (e.g. confidence intervals)
☐	☒	For null hypothesis testing, the test statistic (e.g. *F*, *t*, *r*) with confidence intervals, effect sizes, degrees of freedom and *P* value noted<br>*Give P values as exact values whenever suitable.*
☒	☐	For Bayesian analysis, information on the choice of priors and Markov chain Monte Carlo settings
☒	☐	For hierarchical and complex designs, identification of the appropriate level for tests and full reporting of outcomes
☐	☒	Estimates of effect sizes (e.g. Cohen's *d*, Pearson's *r*), indicating how they were calculated

Our web collection on statistics for biologists contains articles on many of the points above.

Software and code

Policy information about availability of computer code

Data collection	Redcap
Data analysis	All analyses and visualizations were performed in RStudio v2023.06.1.524 with R v4.2.1 using the following packages: tidyverse v2.0.0, haven v2.5.3, Hmisc v5.0.1, ggpubr v0.6.0, geomtextpath v0.1.1, DiagrammeR v1.0.9, broom v1.0.5, broom.mixed v0.2.9.4, ggeffects v1.3.2, gtsummary v1.7.0, ggmosaic v0.3.3, biostat3 v0.1.8, modelr v0.1.11.<br><br>All code used for analysis and figures is available on Github: https://github.com/duke-malaria-collaboratory/imbibe_manuscript

For manuscripts utilizing custom algorithms or software that are central to the research but not yet described in published literature, software must be made available to editors and reviewers. We strongly encourage code deposition in a community repository (e.g. GitHub). See the Nature Portfolio guidelines for submitting code & software for further information.

Data

Policy information about availability of data

All manuscripts must include a data availability statement. This statement should provide the following information, where applicable:

- Accession codes, unique identifiers, or web links for publicly available datasets
- A description of any restrictions on data availability
- For clinical datasets or third party data, please ensure that the statement adheres to our policy

Plasmodium falciparum sequences are available as BioProjectBioProject PRJNA1064031 [https://www.ncbi.nlm.nih.gov/bioproject/PRJNA1064031]. Source data for figures are provided with this paper. Data from human participants in this study are not made available in an open repository due to privacy issues and conditions of IRB approval. Data can be requested from the Principal Investigators (O'Meara and Taylor), who will respond to requests within two weeks. Investigators interested in the dataset will be asked to provide a brief study description/analysis plan. Data will be provided via a secure link. No identifying information will be shared and data recipients will not be permitted to share data with other investigators.

Research involving human participants, their data, or biological material

Policy information about studies with human participants or human data. See also policy information about sex, gender (identity/presentation), and sexual orientation and race, ethnicity and racism.

Reporting on sex and gender	Outcomes are reported by biological sex. Information about sex is based on self-report
Reporting on race, ethnicity, or other socially relevant groupings	Outcomes are not disaggregated by race or ethnicity.
Population characteristics	See Table 1 of the main text
Recruitment	This is a population-based cohort. Households were selected in 5 villages by starting with a random household and enrolling adjacent households in outward radiating circles until 15 households were enrolled in a geographically contiguous cluster. All members of an individual household are eligible to participate
Ethics oversight	The study was approved by the ethical review boards of Moi (2017/36) and Duke (Pro00082000) Universities

Note that full information on the approval of the study protocol must also be provided in the manuscript.

Field-specific reporting

Please select the one below that is the best fit for your research. If you are not sure, read the appropriate sections before making your selection.

☒ Life sciences ☐ Behavioural & social sciences ☐ Ecological, evolutionary & environmental sciences

For a reference copy of the document with all sections, see nature.com/documents/nr-reporting-summary-flat.pdf

Life sciences study design

All studies must disclose on these points even when the disclosure is negative.

Sample size	Sample size was dictated by the number of mosquitoes collected and not selected a priori
Data exclusions	Observations were excluded if we could not amplify the STR profile of the bloodmeal or match it to a cohort member
Replication	All PCR amplifications were done in duplicate with appropriate controls.
Randomization	There was no randomization in this study
Blinding	There was no blinding in this study, as participants were not allocated to different groups.

Reporting for specific materials, systems and methods

We require information from authors about some types of materials, experimental systems and methods used in many studies. Here, indicate whether each material, system or method listed is relevant to your study. If you are not sure if a list item applies to your research, read the appropriate section before selecting a response.

Materials & experimental systems

n/a	Involved in the study	
☒	☐	Antibodies
☒	☐	Eukaryotic cell lines
☒	☐	Palaeontology and archaeology
☐	☒	Animals and other organisms
☒	☐	Clinical data
☒	☐	Dual use research of concern
☒	☐	Plants

Methods

n/a	Involved in the study	
☒	☐	ChIP-seq
☒	☐	Flow cytometry
☒	☐	MRI-based neuroimaging

Animals and other research organisms

Policy information about studies involving animals; ARRIVE guidelines recommended for reporting animal research, and Sex and Gender in Research

Laboratory animals	The study did not involve laboratory animals.
Wild animals	Indoor resting mosquitoes were caught by vacuum aspiration inside buildings of cohort member households. They were transported to the laboratory on ice and sacrificed with chloroform.
Reporting on sex	This study only reports on female Anopheles mosquitoes, as these are the relevant vectors for malaria.
Field-collected samples	For two weeks out of each month, mosquitoes were immediately sacrificed upon arrival to the laboratory, transected (abdomen, head-thorax, wings), and stored for downstream laboratory analyses.<br><br>One week each month, mosquitoes were collected for rearing and were released into household-specific cages inside an insectary maintained at 27±2°C and 80±10% relative humidity. Mosquitoes fed on cotton saturated with 10% sucrose in distilled water for 7 days prior to sacrificing with chloroform. All female Anopheles spp. were morphologically identified, transected, and stored as described above.
Ethics oversight	The study was approved by the ethical review boards of Moi (2017/36) and Duke (Pro00082000) Universities. No separate ethical approval was required to work with mosquitoes.

Note that full information on the approval of the study protocol must also be provided in the manuscript.

Plants

Seed stocks	*Report on the source of all seed stocks or other plant material used. If applicable, state the seed stock centre and catalogue number. If plant specimens were collected from the field, describe the collection location, date and sampling procedures.*
Novel plant genotypes	*Describe the methods by which all novel plant genotypes were produced. This includes those generated by transgenic approaches, gene editing, chemical/radiation-based mutagenesis and hybridization. For transgenic lines, describe the transformation method, the number of independent lines analyzed and the generation upon which experiments were performed. For gene-edited lines, describe the editor used, the endogenous sequence targeted for editing, the targeting guide RNA sequence (if applicable) and how the editor was applied.*
Authentication	*Describe any authentication procedures for each seed stock used or novel genotype generated. Describe any experiments used to assess the effect of a mutation and, where applicable, how potential secondary effects (e.g. second site T-DNA insertions, mosiacism, off-target gene editing) were examined.*
